# Eco-friendly and green synthesis of silver nanoparticles using leaf extract of *Strychnos potatorum* Linn.F. and their bactericidal activities

**DOI:** 10.1007/s13205-014-0272-3

**Published:** 2014-12-31

**Authors:** Srikanth Kagithoju, Vikram Godishala, Rama Swamy Nanna

**Affiliations:** Department of Biotechnology, Kakatiya University, Warangal, 506 009 India

**Keywords:** *Strychnos potatorum*, Leaf extract, Silver nanoparticles, Bactericidal activity, Human pathogenic bacteria

## Abstract

Inspired green synthesis of metallic nanoparticles is evolving as an important branch of nanotechnology. Traditionally these are manufactured by wet chemical methods which require toxic and flammable chemicals. We report for the first time an economic and eco-friendly green synthesis of silver nanoparticles using *Strychnos potatorum* aqueous leaf extract from 3 mM silver nitrate solution. Nanoparticles thus formed are confirmed and characterized by using UV–Vis absorption spectroscopy, SEM and XRD measurements. The XRD and SEM analysis showed the average particle size of nanoparticles as 28 nm as well as revealed their (mixed, i.e., cubic and hexagonal) structure. Further, these green synthesized nanoparticles showed bactericidal activity against multidrug-resistant human pathogenic bacteria.

## Introduction

The development of different eco-friendly and green or bioprocesses for the synthesis of metal nanoparticles with a big surface/volume ratio is evolving into an important branch of nanotechnology, because they exhibit meticulous properties which are useful in different fields, such as electronics, material sciences, medicine, catalysis and photonics at nanoscale. To date, metallic nanoparticles are mostly prepared from noble metals (silver, platinum, gold) (Leela and Vivekanandan 2008). Among the noble metals, silver is the metal of choice in the field of biological systems and medicine (Parashar et al. 2009). Its disinfectant property is being exploited for hygienic and medicinal purposes, such as treatment of mental illness, nicotine addiction and infectious disease like syphilis and gonorrhea (Gulbrason et al. 2000).

Currently, preparation of silver nanoparticles have drawn the interest of researchers due to their diverse properties and uses in different fields, like magnetic, optical polarizability, electrical conductivity (Chang and Yen 1995), catalysis, antimicrobial and antibacterial activities (Baker et al. 2005; Shahverdi et al. 2007), DNA sequencing (Cao et al. 2001), and surface-enhanced Raman scattering (SERS) (Matejka et al. 1992). These nanoparticles show bactericidal effect against both gram positive and negative bacteria. But, nowadays multidrug-resistant gram-negative bacteria are increasing due to their ability to produce β‐lactamases, metallo‐β‐lactamases and carbapenemases and are difficult to treat (Kagithoju et al. 2012) which can possibly be treated with silver nanoparticles.

A number of approaches are available for the synthesis of silver nanoparticles, such as chemical reduction of silver ions in aqueous solutions with or without stabilizing agents (Liz-Marzan and Lado-Tourino 1996), thermal decomposition in organic solvents (Esumi et al. 1990), chemical reduction and photo-reduction in reverse micelles (Pileni 2000; Sun et al. 2001), radiation chemical reduction (Henglein 1993, 1998, 2001), microwave-assisted process (Pastoriza-Santos 2002) and recently via green chemistry route (Begum et al. 2009; Bar et al. 2009). Most of these methods except green chemistry route are expensive and also involve the use of toxic, hazardous chemicals, which may pose potential environmental and biological risks. Since nanoparticles are widely applied to areas of human contact (Jae and Beom 2009), there is a growing need to develop eco-friendly processes for nanoparticles synthesis that do not use toxic chemicals. The use of environmentally benign materials/bio-agents like microorganisms (Klaus et al. 1999; Nair and Pradeep 2002; Konishi and Uruga 2007), enzymes (Wilner et al. 2006), fungus (Vigneshwaran et al. 2007) and plants or plant extracts (Chandran et al. 2006; Jae and Beom 2009) for the synthesis of silver nanoparticles offers numerous benefits of eco-friendliness and compatibility for pharmaceutical and other biomedical applications as they do not use toxic chemicals for the synthesis protocol and can be even easily scaled up for large-scale synthesis. Green synthesis of silver nanoparticles have advantages over the chemical and physical method as it is money-spinning, eco-friendly, one-step method, easily scaled up for large-scale synthesis and does not require high pressure, energy, temperature and toxic chemicals for production (Jain et al. 2010).

Hence, we report the biosynthesis of silver (Ag) nanoparticles using the *Strychnos potatorum* Linn.F. (Loganiaceae) cell-free aqueous leaf extract as bio-reducing agent and their bactericidal activity. *S. potatorum* is commonly known as *Grape Strychnos* or *Clearing nut tree* and is one of the fast disappearing endangered medicinally important forest tree species (Kagithoju et al. 2012, 2013). The seed, besides its bark and root, is used in the Indian traditional systems of medicine for treating various diseases including microbial infections. It is used in Ayurveda for treating the eye and urinary tract infections (Bisset 1974), gonorrhea and kidney troubles in Greek medicine system and for the leucorrhoea, tuberculosis, venereal diseases and acute diarrhea in Siddha medicine (Kirtikar and Basu 1998). Pounded leaves are used to treat watering and aching eyes, leaf decoction is taken to treat epilepsy and cough.

During the present investigation, the unexploited potential of the vulnerable medicinal plant *S. potatorum,* leaf extract is used for nanoparticles preparation and also studied their bactericidal activity against *Staphylococcus aureus* and *Klebsiella pneumoniae*. This method is advantageous over other environmentally benign biological methods like use of bacterial cultures by avoiding elaborate process of maintaining cell cultures. The bactericidal activity of these nanoparticles may even provide a new platform to this plant in nanotechnology and also in development of nanoparticles-based drugs in the treatment of infectious diseases caused by *S. aureus and K. pneumoniae*.

## Materials and methods

### Preparation of leaf extract

*S. potatorum* leaves were collected from the plants available in the premises of Govt. Timber Depot (GTD), Mahadevpur, Karimnagar, Andhra Pradesh, India, during February 2012 and authenticated by the Prof. N. Rama Swamy, Department of Biotechnology, Kakatiya University, Warangal. These leaves were washed under running tap water, treated with 15 % (W/V) Bavistin solution (Fungicide) for 5 min followed by 70 % (V/V) ethanol (1 min). Later, these were washed with sterile distilled water for three times. The leaves were air dried for 5 days and were kept in the hot-air oven at 60 °C for 24–48 h. The leaves were ground to a fine powder and 25 g of leaf powder was boiled in 100 mL of sterile distilled water for 10 min in a 250-mL Erlenmeyer flask. The mixture was then filtered through Whatman No. 4 filter paper and centrifuged at 8,000 rpm for 20 min to get cell-free leaf extract. This extract was stored at 4 °C and used within 1 week.

### Biosynthesis of silver nanoparticles

Silver nitrate was purchased from Sigma-Aldrich, USA. For green synthesis of silver nanoparticles from leaf extract, about 10 mL of leaf extract was added to 90 mL of 3 mM aqueous silver nitrate solution and incubated in a rotary orbital shaker at 150 rpm. The reaction was carried out for a period of 24 h at 25 °C in dark.

### UV–visible spectra analysis

The color change in reaction mixture was recorded through visual observation. The bio-reduction of Ag^+^ ions into Ag^0^ in aqueous solution was monitored by measuring UV–Vis spectrum of the reaction mixture after 5 h by diluting an aliquot of 0.1 mL of sample into 2 mL with deionized water within the range of 350–500 nm at a resolution of 0.5 nm using UV–Vis spectrophotometer (model UV-1800 Shimadzu, Japan), because it has already been reported that the absorption spectrum of aqueous Ag(NO_3_)_2_ only solution exhibited *λ* max at about 220 nm where as silver nanoparticles *λ* max at about 430 nm (Amkamwar et al. 2005).

### Scanning electron microscopic (SEM) observation of silver nanoparticles

To determine the shape and size of nanoparticles, SEM analysis was done by using scanning electron microscope (Model: JOEL-JSM 5600) as per the standard procedure (John and Lonnie 1988). For SEM observation, the residual solution of 50 mL after reaction was centrifuged at 6,000 rpm for 10 min and the resulting suspension was redispersed in 5 mL of deionized water. The centrifugation and redispersing process was repeated 3 times to remove biomass. Later, the sample was directly mounted over the stubs with double-sided carbon conductivity tape and a thin layer of gold coat over the samples were done by using an automated sputter coater (Model: JEOL JFC-1600) for 3 min and scanned under SEM at required magnification.

### X-ray diffraction (XRD) measurement

After obtaining the purified silver nanoparticles by centrifugation and redispersion method, they were freeze dried and structure was analyzed by an X’Pert Pro X-ray diffractometer. The crystallite domain size was calculated from the width of the XRD peaks, assuming that they are free from non-uniform strains, using the Scherrer formula *D* = 0.94*λ*/*β* Cos *θ*, where, *D* is the average crystallite domain size perpendicular to the reflecting planes, *λ* is the X-ray wavelength, *β* is the full width at half maximum (FWHM) and *θ* is the diffraction angle.

### Bactericidal studies

The bactericidal studies were done on multidrug-resistant (MDR) human pathogens *S. aureus and K. pneumoniae* by agar well diffusion method (Perez et al. 1990). The standard pathogenic bacterial strains were procured from the Department of Microbiology, Kakatiya University, Warangal, and used in the present study. The bacterial cultures were revived in Mueller–Hinton broth (Hi-media laboratories, Mumbai, India) at 37 °C for 16–18 h and then preserved at 4 °C for future use. A loop full of culture was inoculated in 10 mL of sterile nutrient broth and incubated at 37 °C for 3–4 h. Turbidity of the culture was standardized to 10^5^ CFU with the help of Standard Plate Count and turbidometer. Petri plate containing 20 mL nutrient agar medium was inoculated with 0.1 mL of 18-h-old bacterial suspension culture by spread plate method to form lawn cultures. The freeze-dried silver nanoparticles were redispersed in sterile deionized water aseptically. Various concentrations viz., 5, 10, 15 and 20 μg mL^−1^ of the solution with nanoparticles were added into the 6 mm diameter well and incubated for 24 h at 37 °C. 30 μg mL^−1^ streptomycin sulphate (Hi-media laboratories, Mumbai, India) was used as positive control. To study the bactericidal activity of nanoparticles, the diameter of the inhibition zone formed around the well is measured in mm.

## Results and discussion

**Fig. 1:**
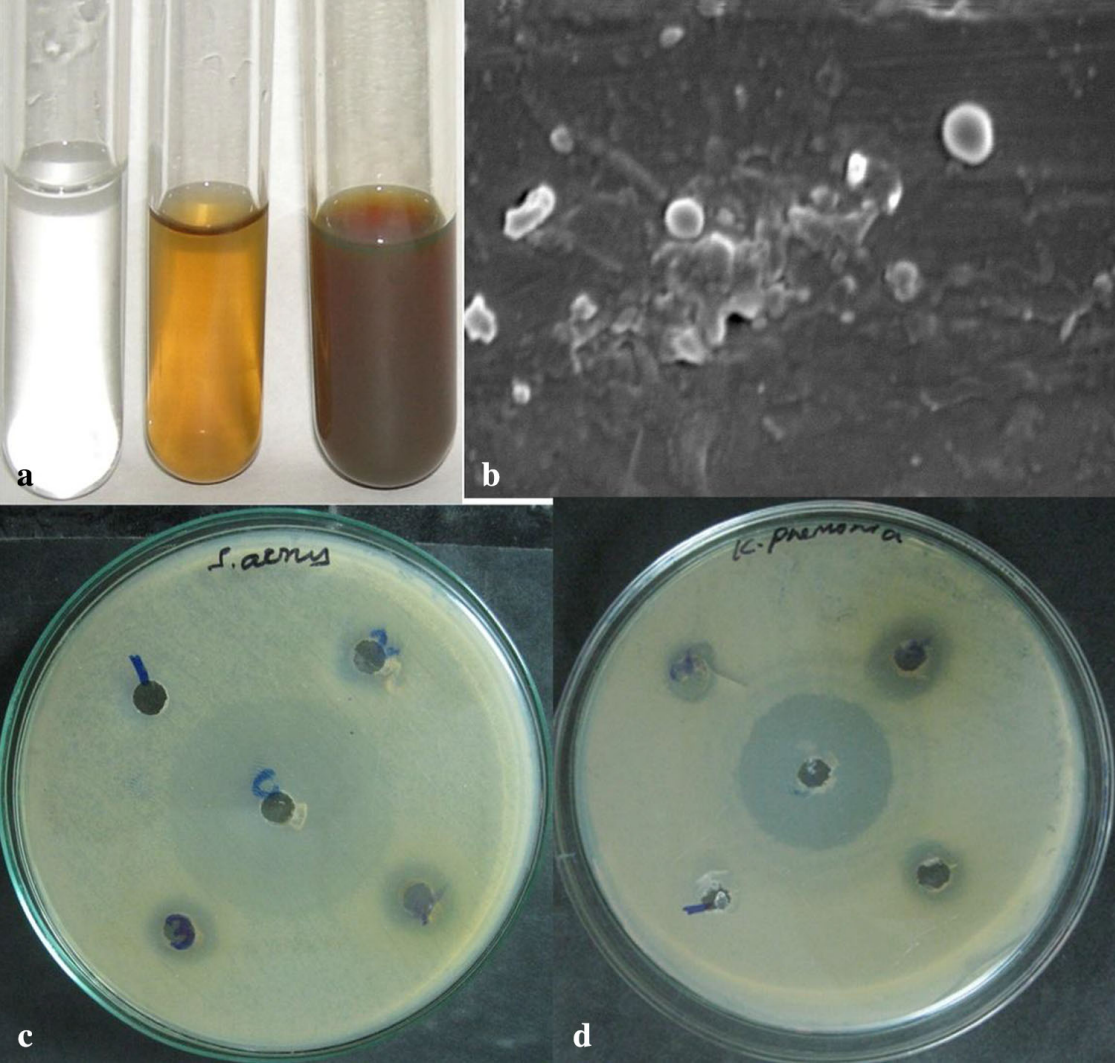
Green synthesis of silver nanoparticles using *S. potatorum* leaf extract: **a** color change in 3 mM Ag(NO_3_)_2_ solution after treatment, *left tube* 3 mM Ag(NO_3_)_2_ solution, *middle tube* Ag(NO_3_)_2_ solution with leaf extract immediately after addition, *last tube* after 24 h. **b** SEM analysis of silver nanoparticles. **c**, **d** Bactericidal activity of silver nanoparticles on *S. aureus* and *K. pneumonia* at different concentrations *1st well* 5 μg mL^−1^, *2nd well* 10 μg mL^−1^, *3rd well* 15 μg mL^−1^ and *4th well* 20 μg mL^−1^ (*middle well* *c* control)

**Fig. 2:**
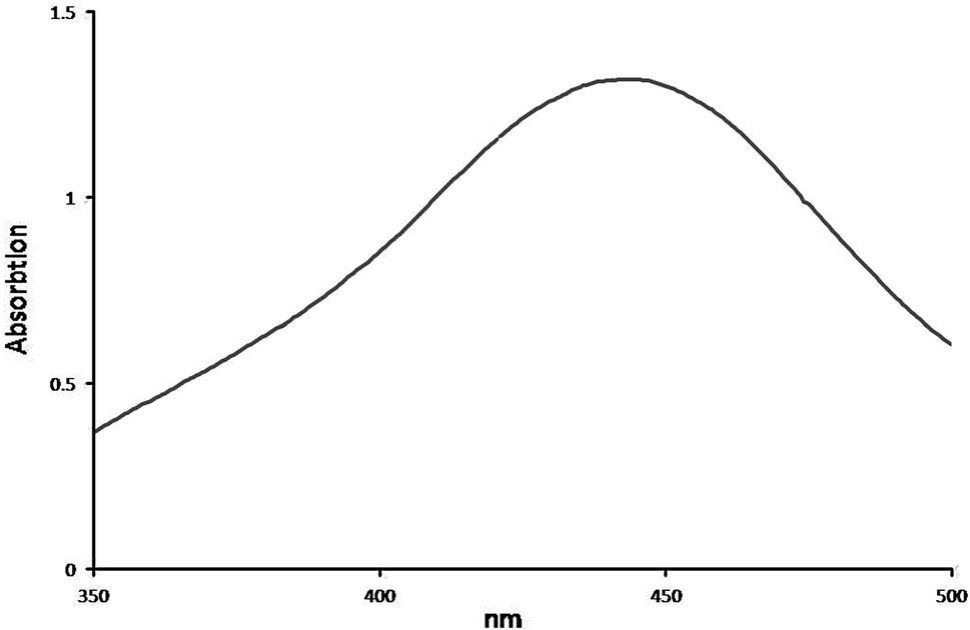
UV–Vis absorption spectra of green synthesized silver nanoparticles in the leaf extract of *S. potatorum*

Reduction of silver ions into silver particles and extra-cellular green synthesis of silver nanoparticles occurred during the exposure of *S. potatorum* leaf extract to 3 mM aqueous silver nitrate solution for this 50 mL of 3 mM silver nitrate solution was taken as the starting material. After addition of leaf extract to silver nitrate solution, a visible color change (pale yellow) was observed and the intensity of the color enhanced to form dark brown color (Fig. 1a) with increase in incubation period. The appearance of dark yellowish brown color in reaction mixture indicated the formation of silver nanoparticles. The color development is due to excitation of surface plasmon vibration in the metal nanoparticles (Mulvaney 1996) and is measured by UV–Vis spectroscopy, which gives an indirect confirmation for formation of silver nanoparticles. The time duration for change in color varies from system to system. The biosynthesis of silver nanoparticles in the leaf extract of *S. potatorum* was observed within 30 min. This had been confirmed by measuring the UV–Vis spectrum (Fig. 2) of the reaction mixture. In the present study, the UV–Vis spectrum of colloidal solution of the nanoparticles showed the absorbance peak at 430 nm which is a characteristic feature of silver nanoparticles and the broadening of the peak indicated the particles are polydispersed. Similar results were also observed in *Geranium* leaf extract (Shankar et al. 2003).

**Fig. 3:**
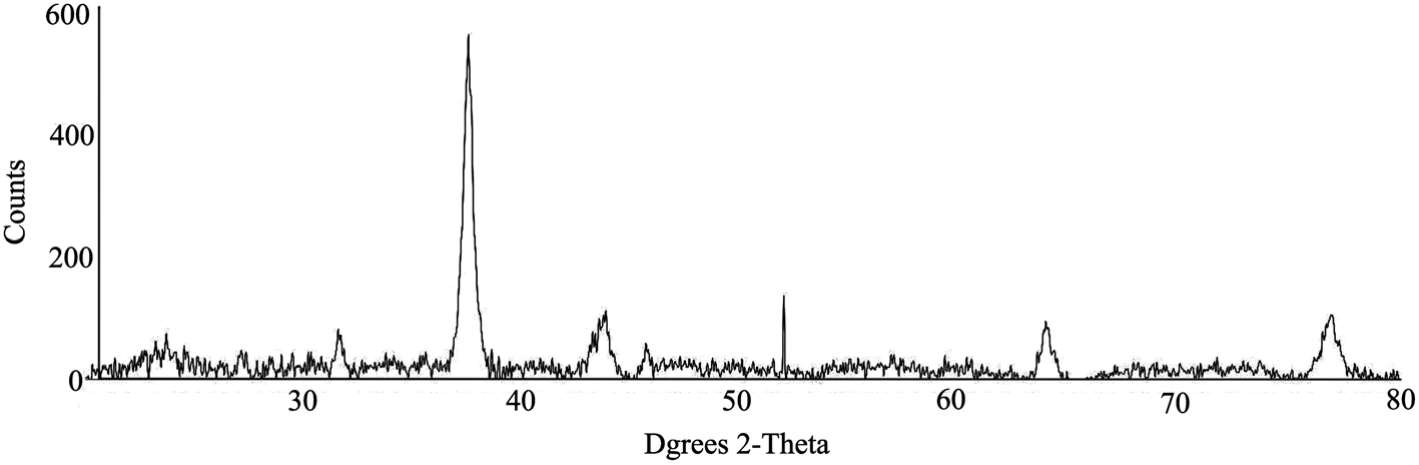
XRD pattern of green synthesized silver nanoparticles in the leaf extract of *S. potatorum*

The biosynthesis of nanoparticles in *S. potatorum* leaf extract was further confirmed by the characteristic peaks observed in the XRD pattern (Fig. 3) and the structural view under the SEM (Fig. 1b). The XRD pattern showed four intense peaks in the whole spectrum of 2*θ* value ranging from 20 to 80. Average size of the particles synthesized was 28 nm ranging from 20 to 62 nm with cubic and hexagonal shape. The typical XRD pattern showed that the sample contains a mixed phase (cubic and hexagonal) silver nanoparticles. The average estimated particle size of this sample was 28 nm derived from the FWHM.

SEM image (Fig. 1b) showed relatively spherical, individual as well as a number of aggregates of nanoparticles with diameter ranging from 18 to 60 nm. Similar results were also observed in plant extracts of *Aloe vera* (Chandran et al. 2006), *Emblica officinalis* (Amkamwar et al. 2005) and *Carica papaya* (Jain et al. 2009).

Toxicity study of nanoparticles on pathogens opened new avenue for nanotechnological applications in medicine. The green synthesis of silver nanoparticles using medicinal plants was found to be toxic against most of the pathogens (Savithramma et al. 2011). In the present investigation, nanoparticles synthesized by *S. potatorum* leaf extract were found to be effective against MDR human pathogenic bacteria *S. aureus and K. pneumoniae* at a concentration of 20 µg mL^−1^ (Fig. 1c, d). Silver nanoparticles showed maximum inhibition zone in *K. pneumoniae* (10 mm) compared to *S. aureus* (8 mm). Similar observations were found in *Euphorbia hirta* (Elumalai et al. 2010) and *Boswellia ovalifoliolata* and *Shorea tumbuggaia* (Savithramma et al. 2011). Thus, the present investigation supports the role of *S. potatorum* in biosynthesis of silver nanoparticles and their capability of rendering the bactericidal activity.

## Conclusion

The present investigation revealed that the *S. potatorum* leaf extract would be a good source for green synthesis of silver nanoparticles. The bio-reduction of silver ions by leaf extract was confirmed by the brown color formation within 20–30 min. Bactericidal activity of these silver nanoparticles against MDR gram positive and negative bacteria *S. aureus* and *K. pneumoniae* confirmed that the silver nanoparticles are capable of rendering antibacterial activity and also strengthening the biomedicine value of the plant. This eco-friendly and green synthesis of silver nanoparticles is simple and convenient to handle and most advantage and economic. The bactericidal study of these nanoparticles may play a vital role in the treatment and invention of new drugs against diseases/infections caused by *S. aureus* (lower respiratory tract infections, surgical site infections, nosocomial bacteremia, pneumonia, cardiovascular infections, etc.) and diseases caused by *K. pneumonia* (urinary tract infections, pneumonia, septicemias and soft tissue infections, etc.). The bactericidal studies of these nanoparticles against some other MDR bacteria are under investigation.
